# Local and Global Context-Enhanced Lightweight CenterNet for PCB Surface Defect Detection

**DOI:** 10.3390/s24144729

**Published:** 2024-07-21

**Authors:** Weixun Chen, Siming Meng, Xueping Wang

**Affiliations:** 1 The Information Engineering Institute, Guangzhou Railway Polytechnic, Guangzhou 510430, China; 2 Key Laboratory of Equipment Safety and Intelligent Technology of Guangzhou Rail Transit System, Guangzhou 510430, China; 3 The Science and Engineering College of Information, Hunan Normal University, Changsha 410081, China

**Keywords:** PCB surface defect detection, lightweighting, CenterNet, PANet, two-branch

## Abstract

Printed circuit board (PCB) surface defect detection is an essential part of the PCB manufacturing process. Currently, advanced CCD or CMOS sensors can capture high-resolution PCB images. However, the existing computer vision approaches for PCB surface defect detection require high computing effort, leading to insufficient efficiency. To this end, this article proposes a local and global context-enhanced lightweight CenterNet (LGCL-CenterNet) to detect PCB surface defects in real time. Specifically, we propose a two-branch lightweight vision transformer module with local and global attention, named LGT, as a complement to extract high-dimension features and leverage context-aware local enhancement after the backbone network. In the local branch, we utilize coordinate attention to aggregate more powerful features of PCB defects with different shapes. In the global branch, Bi-Level Routing Attention with pooling is used to capture long-distance pixel interactions with limited computational cost. Furthermore, a Path Aggregation Network (PANet) feature fusion structure is incorporated to mitigate the loss of shallow features caused by the increase in model depth. Then, we design a lightweight prediction head by using depthwise separable convolutions, which further compresses the computational complexity and parameters while maintaining the detection capability of the model. In the experiment, the LGCL-CenterNet increased the mAP@0.5 by 2% and 1.4%, respectively, in comparison to CenterNet-ResNet18 and YOLOv8s. Meanwhile, our approach requires fewer model parameters (0.542M) than existing techniques. The results show that the proposed method improves both detection accuracy and inference speed and indicate that the LGCL-CenterNet has better real-time performance and robustness.

## 1. Introduction

Printed circuit boards (PCBs) are the cornerstone of most electronic products. Any manufacturing defect on a PCB can lead to fatal problems in electronic products [1]. Therefore, it is critical to design effective approaches for identifying surface defects. Traditionally, manual visual inspection is widely used for PCB defect detection, which is labor-intensive and inefficient [2]. In addition, as the global demand for electronics continues to grow, only a limited percentage of samples are detected, and faulty items can readily be combined with products exempt from detection [3]. Meanwhile, to satisfy the market need for increasingly complicated electronic circuit manufacturing procedures, bare PCBs have become more complex and highly integrated [4]. Traditional manual inspection is harder to recognize when the density increases. Thus, significant efforts have been made to automate the inspection by utilizing high-resolution CCD or CMOS sensors [5]. With the development of intelligent sensors, PCB image sample collection has become easier [6]. Based on these images samples, computer vision techniques will help to efficiently estimate and predict product quality [7].

Visual inspection methods can be classified into three types: traditional imageology algorithms [8], machine learning-based algorithms [9], and deep learning-based algorithms [10]. Traditional imageology identifies defects using generic visual properties such as texture, edge contour, and contrast; however, these methods have some shortcomings such as fixed application scenarios and poor robustness. Conversely, machine learning-based methods, such as support vector machines (SVMs) [11] and decision trees [12], have been widely used in PCB surface defect detection, improving the accuracy and efficiency of defect detection to some extent and allowing detection of more types of defects. However, these methods rely heavily on hand-crafted features, making it difficult to distinguish and locate small defects in complex texture interference on PCB surfaces [1]. Recently, with the deployment of end-to-end deep learning algorithms, the limitations of the aforementioned methods, such as dependency on a priori template, have been partially solved [1].

Convolutional neural networks (CNNs) are beneficial for extracting image information and are not dependent on manual adjustments to the parameters [13]. The topic of surface defect detection has seen the use of many deep learning-based object detection techniques, which may be divided into two categories based on how the object is localized: one-stage methods [14,15] and two-stage methods [16,17,18]. While two-stage algorithms are capable of effectively identifying defects, their detection efficiency is limited, their training is complicated, and their model volume is substantial. As a result, they are not suitable for PCB surface defect detection in industrial scenarios that require lightweight and rapid detection [19]. One-stage techniques streamline the network architecture and training procedures, increase detection efficiency, and strike a balance between the network parameters and detection performance required for real-time inspection in the modern industry. CenterNet is a one-stage object detection network that has significant advantages over other models in terms of deployment, speed, and small target detection [19]. Meanwhile, CenterNet is an anchor-free algorithm. Compared with anchor-based algorithms [20,21,22], the network structure is simpler, and the inference speed is faster.

**Figure 1:**
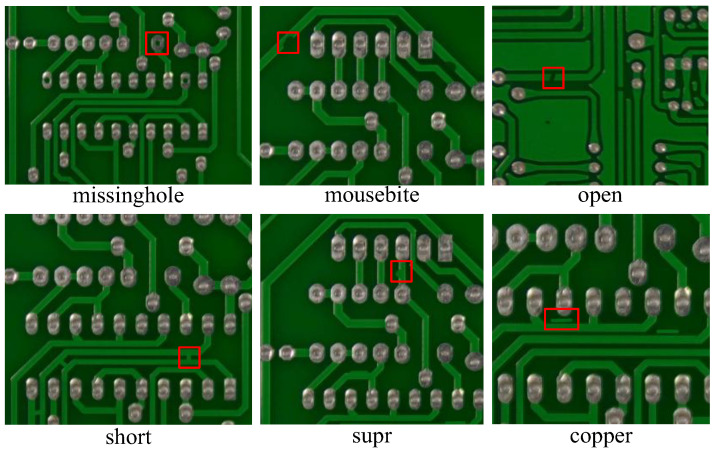
Six different kinds of PCB defects.

**Figure 2:**
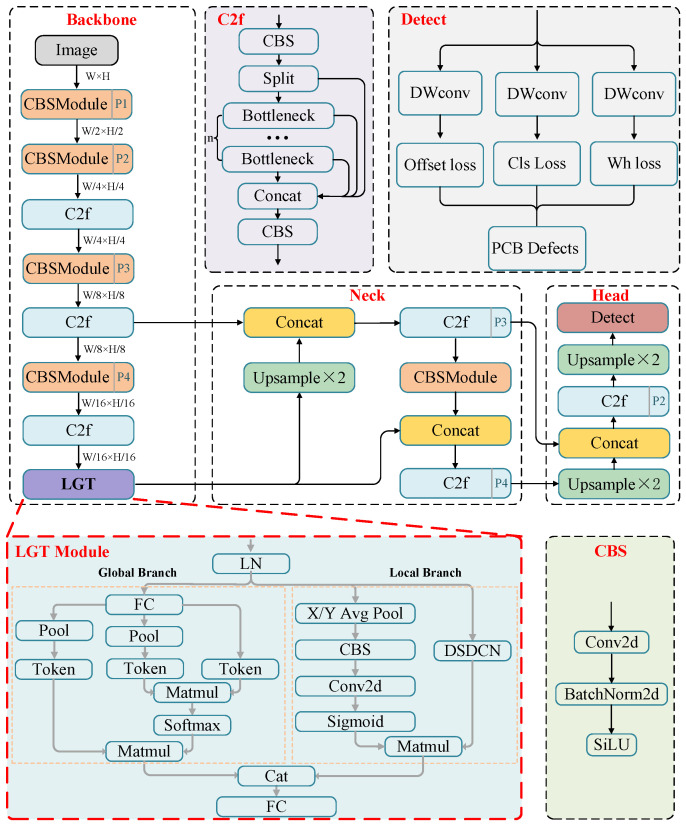
The overall network architecture of the proposed local and global context-enhanced lightweight CenterNet.

In this article, a local and global context-enhanced lightweight CenterNet (LGCL-CenterNet) is proposed to effectively detect six common PCB defects [23] including missing hole, mouse bite, open circuit, short circuit, spur, and spurious copper, as shown in Figure 1. Specifically, a lightweight backbone network based on Darknet-53 is first created to efficiently extract multiscale features by removing the down-sampled feature maps (P5) at 1/32 of the original image size, which reduces the computational complexity and number of parameters of the model. Then, we introduce a two-branch lightweight vision transformer module with local and global attention (LGT) to extract high-dimension features and leverage context-aware local enhancement after the backbone network. After that, a Path Aggregation Network (PANet) feature fusion structure is introduced to mitigate the loss of shallow features. Finally, a lightweight prediction head with depthwise separable convolutions [24] is designed to output final results, which further compresses the computational complexity and parameters while maintaining the detection performance. The overall network architecture is outlined in Figure 2.

The following is a summary of the main contributions of this paper.

1. Design a two-branch lightweight real-time visual transformer block (LGT block) for efficient fusion and processing of local and global context information to improve the feature extraction capability.

2. Design a lightweight backbone network and head network to achieve efficient defect detection, which greatly reduces the number of parameters and FLOPs when compared to the original CenterNet network.

3. The experimental result shows that the proposed algorithm has better detection performance than existing mainstream target detection algorithms. Specifically, the CenterNet model using ResNet18 as the backbone network has about 14.128M parameters while the proposed algorithm has only about 0.542M parameters, yet the mAP@0.5 is improved by about 2%.

The remainder of this paper is arranged as follows. Section 2 summarizes the study on detecting PCB surface defects. The proposed method is detailed in Section 3. Section 4 reports the experimental results as well as their analysis. Finally, in Section 5, we review and assess the whole research and discuss future work.

## 2. Related Work

Due to the high cost and easy visual fatigue of the visual inspection method and the fact that the electrical measurement method can only detect the electrical function defects of the PCB, its detection range and capabilities are restricted. In recent years, many studies have been conducted on the computer vision-based PCB surface defect detection technique, and it is expected to replace the traditional measurement method [25]. These approaches are mainly divided into traditional visual inspection algorithms, machine learning-based algorithms, and deep learning-based algorithms.

### 2.1. Traditional Visual Inspection Algorithms

For PCB surface defect detection based on traditional methods, an effective similarity measuring technique was presented [8] to deal with scene and reference images that have notable differences in illumination and noise. The technique described in [26] compares the whole Fourier spectrum between the inspection image and the template to identify defects in images, such as printed circuit boards or integrated circuit dies, found in the electronics industry. To precisely identify the defect location and type, a real-time PCB automated defect identification approach based on SURF characteristics and morphological operations is proposed [27]. The purpose of the image subtraction approach [28] is to identify and categorize PCB defects. Furthermore, a fast surface detection technique is designed in [29] based on sparse representation. Although classical detection algorithms offer some detecting effects, they often have strict application limitations and cannot meet industry demands for robustness and real-time performance.

### 2.2. Machine Learning-Based Algorithms

Algorithms built on machine learning [30,31] first extract the pattern of input data and then feed it into classifiers to predict defects. Ref. [32] extracts features based on wavelet transform, followed by a k-nearest neighbors classifier to classify the part under test. Support vector machine (SVM) is utilized [33] to classify defects based on the local binary pattern features as well as the oriented gradients histogram of the extracted data. The method in [34] designs a framework for detecting PCB surface defects utilizing machine learning techniques. Histogram equalization, the Laws filter, and the Sobel filter are used to extract features and artificial neural networks and SVM are explored for fabric texture classification [35]. Although the weak robustness of traditional detection algorithms has been partially addressed by machine learning-based detection algorithms, the hand-crafted feature extraction approaches rely on complicated expert knowledge and are easily impacted by external noise, resulting in poor generalization performance. In addition, it is difficult to locate manufacturing defects using machine learning techniques.

### 2.3. Deep Learning-Based Algorithms

Recently, the use of deep learning-based techniques has grown in popularity due to the quick advancements in computer efficiency and sensor technologies. These methods do not require carefully designed feature extraction methods and can adaptively extract multiscale information from input images, resulting in greater robustness and performance [3]. Numerous deep learning-based object identification techniques [36,37] have also been used for defect detection. These pipelines may be broadly divided into two categories based on the object localization method including both one-stage and two-stage approaches [4].

Regarding the study of detection algorithms based on two-stage target detection networks, Hu et al. [38] introduced the feature pyramid network (FPN) to improve the small target detection capability of the original Faster RCN. Atrous spatial pyramid pooling (ASPP)-balanced FPN (ABFPN), an improved multiscale feature fusion technique, is designed to improve tiny object identification performance [39]. By including a cost-sensitive adjustment layer in the conventional ResNet, the authors in [20] propose the cost-sensitive residual convolutional neural network (CSResNet), which successfully balances class distribution and distinguishes between true defects and fake defects in PCB identification. Road fractures can be detected using a combination training technique that combines Faster R-CNN and Mask R-CNN [40]. Although the two-stage target detection algorithm achieves better detection performance, it is unsuitable for identifying PCB surface defects in production environments demanding lightweight and real-time detection due to its complexity of training stages, slow detection speed, and large model size.

The two-stage technique is slower because it splits the object identification issues into two steps: extracting regions of interest (RoIs) and then classifying and regressing the RoIs. Experts and academics propose one-stage object detection methods by reducing the laborious localization processes and combining the localization and classification of the detected objects into a single network, hence improving the inference speed and simplifying the network structure and training stages. As a result, these techniques can produce accurate and quick outcomes. For example, Kang et al. [21] construct a tiny target prediction feature layer module to enhance the perception ability of small target characteristics and design a multi-layer SSD for PCB defect detection. An effective tiny defect detection network with a parallel convolution module, serial convolution module, and feature fusion module is proposed [22], which achieves a desirable trade-off between speed and accuracy. By combining the advantages of transformer and convolutional networks, the authors in [1] present an improved YOLOv5 method to make use of global dependencies and location information for PCB defect identification. A global contextual attention-augmented YOLO model with ConvMixer prediction heads (GCC-YOLO) is proposed to mitigate missed and erroneous detection [4]. Focal loss is designed by RetinaNet [41,42,43] to address the severe foreground–background class imbalance of one-stage detectors. It can outperform many current state-of-the-art two-stage detectors in terms of accuracy while matching the speed of the one-stage detectors. The majority of one-stage object identification algorithms contain a large number of parameters that make them unsuitable for industrial applications, despite the fact that they can generally achieve acceptable detection accuracy. Lightweight deep learning algorithms are attracting widespread attention, especially in defect detection in the industrial production field. Zhang et al. [44] propose an efficient lightweight CNN model for surface defect detection of industrial productions, incorporating an inverse residual architecture with coordinate attention and a multi-scale strategy. Hu et al. [45] propose Sim-YOLOv5s, an efficient defect-detection model for lithium battery steel shells, utilizing a fast spatial pooling pyramid structure and attention mechanism. A lightweight model, STMS-YOLOv5 [46], is proposed for gear surface defect detection, utilizing ShuffleNetv2 backbone, transposed convolution upsampling, and max efficient channel attention.

The aforementioned methods almost all use the anchor-based flowchart [20,21,22], which needs to manually set suitable anchor boxes for training an excellent anchor-based object detection model. In addition, to guarantee detection performance, these approaches need a high number of anchors, but using more anchors results in a complex architecture and slow inference [19]. Based on the problems of the above algorithm, this paper proposes an anchor-free object detection algorithm, local and global context-enhanced lightweight CenterNet (LGCL-CenterNet), for efficient PCB defect detection, which has lightweight model volume and lower model complexity but achieves better PCB defect detection accuracy compared with the state-of-the-art approaches. Meanwhile, the proposed approach eliminates the requirement for anchor boxes and overcomes the disadvantages of anchor-based techniques, so our method can be more easily deployed to the manufacturing line.

## 3. Methodology

To reduce the computational resources required to deploy deep learning-based models to the manufacturing line and overcome the drawbacks of anchor-based approaches, this paper proposes a lightweight and anchor-free method, LGCL-CenterNet, based on CenterNet and attention architectures [4,47,48]. In this section, the basic framework of CenterNet and used attention architectures are introduced first. Then, the overall framework is detailed.

### 3.1. Review of CenterNet

Although CenterNet [19] is a one-stage keypoint-based object detection technique, it can achieve similar performance to the two-stage detectors. CenterNet uses a backbone network and three branch networks (head network) to complete target detection. The backbone network is applied to obtain multi-scale image representation, and the branch networks are used to predict the bounding box and category information of the target. Specifically, CenterNet predicts the center point of the target as well as the offset from the center point to the target bounding box to achieve target detection. In addition, CenterNet achieves good performance on multiple target detection datasets, especially in small and dense target detection, which is well suited to the detection of PCB defects [19]. Overall, CenterNet has received widespread attention and application in the field of target detection due to its simple and efficient design and excellent performance.

### 3.2. Attention Architectures

Attention mechanism is critical in the field of deep learning, allowing neural networks to process input data more flexibly and improving a network’s ability to understand and express the input. In this paper, we design a two-branch lightweight vision transformer module with local and global attention based on bi-level routing attention [47] and coordinate attention [48], which will be briefly introduced next.

Bi-Level Routing Attention [47]: This uses two-layer routing to provide more flexible computational allocation and content awareness. It achieves good performance and high computational efficiency by query—adaptively focusing on a small subset of the most relevant tokens without attracting the attention of other irrelevant tokens.

Coordinate Attention [48]: This provides a new idea to dynamically adjust the network’s attention distribution based on the spatial location of the features. This approach can help the network to better focus on the important regions in the image, enhancing the effectiveness and accuracy of feature extraction.

### 3.3. The Network Architecture and Loss Function

This paper proposes a one-stage object detection model, LGCL-CenterNet, shown in Figure 2. There are three components including the head, neck, and backbone. The backbone is a combination of the CNN and transformer, which produces the overall features of images. The neck, a Path Aggregation Network (PANet), is used to enhance informative representation by fusing multi-scale image features from the backbone. The processed features are fed into the prediction layer, which then outputs the final coordinates of the bounding box and the class of the objects.

**Figure 3:**
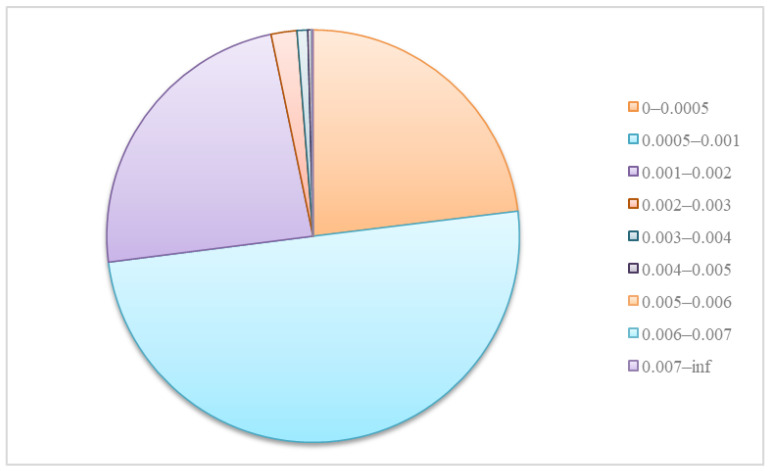
Ratio of PCB defect bounding box area to total image area.

Backbone: In this paper, the backbone network is the Darknet-53 proposed in YOLOv8, which is based on the CSP (Cross Stage Partial) structure and C2f module. Meanwhile, we count the bounding box area of manual annotation in the HRIPCB dataset [49], as shown in Figure 3. It can be seen that the defective portion of the PCB represents only a very small portion (almost less than 2‰) of the total image area. Therefore, we remove the downsampled feature maps (P5) at 1/32 of the original image sizes in the backbone network, because continuous downsampling will lead to missed detection of PCB defects. In addition, it also reduces the parameters as well as memory consumption.

After the backbone network, to efficiently extract image global and local information for PCB defect detection, the YOLO series uses various Spatial Pyramid Pooling (SPP) to further aggregate the features extracted from the backbone network. However, SPP can be computationally expensive, especially when dealing with large feature maps or a large number of spatial bins. This can lead to increased memory and processing requirements, which may limit its applicability in real-time or resource-constrained scenarios. Therefore, this paper designs a two-channel efficient and lightweight visual transformer module (LGT module) for global as well as local information extraction, which is detailed in Section 3.4.

Neck: The backbone network extracts multiscale features, which are then processed by the neck network. It serves to spatially integrate and adjust the feature map to provide more accurate target localization and classification information.

In this paper, the neck network uses PANet (Path Aggregation Network) [50], which is able to integrate multi-scale features and contextual information from different network layers in a systematic and efficient manner. It consists of two main components: feature pyramid network (FPN) and top–down pathway. FPN is the basic component of PANet, which constructs multi-scale feature pyramids by adding lateral connections to the backbone network. The top–down pathway facilitates the propagation of high-level semantic information to lower layers, enabling the network to refine and enrich the representations with contextual information, thus improving the accuracy of target detection.

Predictive Head: Predictive head is designed to transform the feature mapping output from the neck network into the location, category, and other attributes of the target. After PANet, we get the features in P4 and P3 dimensions; to get rich feature representation for PCB defect detection, we first upsample the P4 to P3 dimensions and then use the C2f module to fuse the features. After that, the fused feature is fed into the upsampling layer to generate a higher-resolution feature map for improving the detection of small targets. In other words, the output stride is 2, which is different from the default settings in the literature [19]. In addition, depth separable convolution (DWconv) is used to replace normal convolution in the original CenterNet to reduce the number of parameters and FLOPs. Finally, we follow the CenterNet [19] to detect an object by the center point of its bounding box. The keypoint feature at the center is used to infer the bounding box size and other object attributes.

Specifically, the peaks of each category in the heatmap are extracted independently to obtain center points. For each center point, the bounding box location is (x+δx−w/2, y+δy−h/2, x+δx+w/2, y+δy+h/2), where (x,y) is the detected center point, (δx,δy) is the offset prediction, and (w,h) is the size prediction.

Loss Function: we train the proposed network following [19]. For each ground truth center point, it is splat onto a heatmap using a Gaussian kernel. The training objective is a penalty-reduced pixel-wise logistic regression with focal loss [51]. A local offset for each keypoint is predicted to recover the discretization error caused by the output stride, which is trained using an L1 loss. By the way, this offset prediction is shared for all classes. In addition, L1 loss is also used for size prediction.

### 3.4. Local and Global Context-Enhanced Lightweight Module

To integrate the features of the backbone network, inspired by the Clo block designed in [52], a two-channel efficient and lightweight visual transformer module (LGT module) is designed to extract global and local information.

For the local branch, we design a local high-frequency information extraction module based on coordinate attention (CA) [48]. Specifically, this proposed module first aggregates input features $X\in {\mathbb{R}}^{H\times W\times C}$ in vertical and horizontal directions using two one-dimensional global pooling kernels, (H,1) and (1,W), into two separate direction-aware feature mappings, respectively. Consequently, the output of the *c*-th channel at height h or width w can be expressed as:

(1) $$z_{c}^{h}=\frac{1}{W}\sum_{0\leq i<W}x_{c}(h,i),\;z_{c}^{w}=\frac{1}{W}\sum_{0\leq j<H}x_{c}(j,w)$$

After being embedded with orientation-specific information, these two feature maps, each representing remote dependencies of the input feature maps along a single spatial direction, are encoded into two attention maps, respectively. Then, we concatenate the aggregated feature maps generated by Equation (1) and send them to a shared 1×1 convolution, $F_{1}$.

(2) $$f=\delta (F_{1}([z^{h},z^{w}]))$$

where δ is a non-linear activation function. Moreover, $f\in {\mathbb{R}}^{C\times (H+W)}$ is split into two distinct tensors, $f^{h}\in {\mathbb{R}}^{C\times H}$ and $f^{w}\in {\mathbb{R}}^{C\times W}$, along the spatial dimension. To acquire the attention weights $g^{h}\in {\mathbb{R}}^{C/2\times H}$ and $g^{w}\in {\mathbb{R}}^{C/2\times W}$, two additional 1×1 convolutional transformations, $F_{h}$ and $F_{w}$, are used to convert $f^{h}$ and $f^{w}$ independently, whose channels reduce to half of the input:

(3) $$g^{h}=\delta (F_{h}(f^{h})),\;g^{w}=\delta (F_{w}(f^{w}))$$

**Figure 4:**
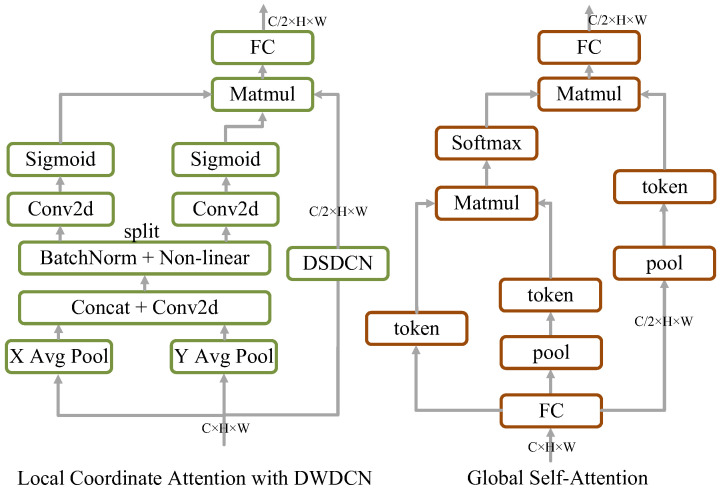
The structure of local coordinate attention and global self-attention.

Consequently, the location information can be preserved in the produced attention maps. In addition, a convolution, $F_{d}$, is used to extract high-frequency features on the input feature maps. To highlight the representation of the region of interest, both attention maps are multiplied by the feature, $F_{d}$,

(4) $$y=F_{d}(x)\times g^{h}\times g^{w}$$

and the structure is shown in Figure 4. The advantages of the proposed local branch are as follows. Firstly, it can capture orientation-aware and location-sensitive information as well as cross-channel features, which improves the ability of the proposed model to more precisely locate and diagnose PCB defects. Second, this module is more flexible and has a small number of parameters, which can be easily applied to real production lines.

For the global feature extraction branch, this paper does not use vanilla attention [53], and the proposed method uses a dynamic, query-aware sparse attention mechanism [47] to suit the real-time demands, whose key idea is to divide the queries and keys into *N* regions, $Q^{r},K^{r}\in {\mathbb{R}}^{N\times C}$, and then use an adjacency matrix, $A^{r}\in {\mathbb{R}}^{N\times N}$, to filter out the majority of the irrelevant key-value pairs at the rough region lever, which is calculated via matrix multiplication based on $Q^{r},K^{r}$,

(5) $$y=F_{d}(x)\times g^{h}\times g^{w}$$

**Figure 5:**
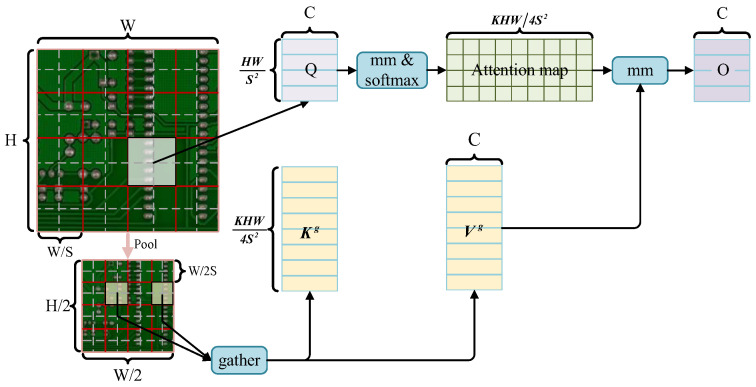
Sparse attention is used to skip computations in the most irrelevant region, and pooling is used to downsample the key and value to reduce FLOPs.

After that, only top-k connections that are relevant to the current query regions (Token) are used to generate the attention map,

(6) $$I^{r}=topkIndex(A^{r})$$

that enables more flexible computational allocation as well as content awareness, and thus it performs well and uses little computing power, particularly in intensive prediction tasks. Token-to-token attention can be applied based on the region-to-region routing index matrix, $I^{r}$. Furthermore, K and V are downsampled to reduce FLOPs, which helps the model capture global information efficiently, as shown in Figure 5.

(7) $$\begin{array}{l} K^{g}=gather(pooling(K),I^{r})\\ V^{g}=gather(pooling(V),I^{r}) \end{array}$$

where $K^{g},V^{g}\in {\mathbb{R}}^{N\times \frac{kHW}{4N}\times C}$ are gathered key and value tensors. We can then apply attention to the gathered key-value pairs as:

(8) $$O=Attention(Q,K^{g},V^{g})$$

Finally, a simple method is used to fuse the outputs of local and global branches. This dual-branch structure allows the LGT module to capture both local and global information for PCB defect detection.

## 4. Experiment

### 4.1. Experimental Data

In this section, the publicly available HRIPCB dataset is used to validate the performance of the proposed method [49]. The six primary manufacturing defects—missing hole (Mh), mouse bite (Mb), open circuit (Oc), short circuit (Sh), spur (Sp), and spurious copper (Sc)—are annotated on 1386 images in the dataset; Figure 1 illustrates the various defect categories. The training set, testing set, and validation set are randomly generated for the experiments with a ratio of 8:1:1.

### 4.2. Data Enhancement and Training Parameters

The proposed algorithm is implemented using Pytorch(v2.3.1). Due to the small amount of PCB data, the proposed algorithm is first pre-trained using the coco dataset [54], then fine-tuned using PCB data. Moreover, in order to train a more robust and accurate model, this paper uses various data enhancement strategies.

Data Enhancement: Data enhancement is especially important for our current task with low data volume. Therefore, this paper uses various image enhancement techniques such as flipping, rotation, and Mosaic to increase the diversity of training data. These methods can effectively improve the performance and robustness of the proposed method. For Mosaic, due to the great difference between the generated images and the real data, this data enhancement method is only used in the first 70% of the iteration steps of training.

Training: We train the proposed algorithm using a single Tesla A100 GPU with a batch size of eight. The resolution of the input image is 3008 × 1568. The maximum number of iterative epochs is 600, the initial learning rate is 5 ×10^−4^, and the final learning rate is 5 ×10^−6^ using a cosine learning rate decay strategy, and the optimizer uses SGD. The images are normalized before entering the network, thus speeding up the convergence of the network.

### 4.3. Experimental Metrics and Experimental Results

Evaluation Metrics: The evaluation measures utilized in this paper include precision (*P*), recall (*R*), average precision (*AP*), mean average precision (*mAP*), and model size. Precision is the percentage of predicted positive samples to all predicted samples. Recall is the percentage of predicted positive samples to total positive samples. Since prediction boxes are used to local potential defects, Intersection over Union (*IoU*) is used to determine whether the prediction boxes are the positive sample. The *IoU* threshold was set to 0.5 in this experiment. This indicates that the prediction box is a positive sample when the overlap ratio is above 0.5. Their calculations are shown in Equations (9)–(11).

(9) $$IoU=\frac{Area\;of\;overlap}{Area\;of\;union}$$

(10) Precision=TP/(TP+FP)

(11) Recall=TP/(TP+FN)

The following formulae can be used to determine average precision (*AP*) and mean average precision (*mAP*),

(12) $$AP=\int_{0}^{1}P(R)\;dR$$

(13) $$mAP=\frac{\sum_{j=1}^{S}AP(j)}{S}$$

*S* represents the number of all categories.

**Figure 6:**
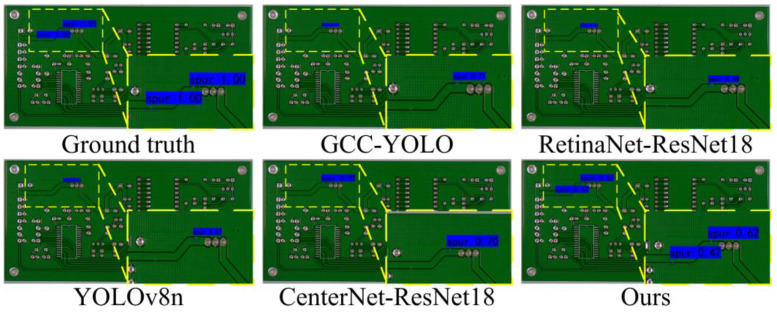
Detection results of different object detection algorithms. More detection results of the other defects can be found in the Supplementary Materials.

Experimental Results: In this study, we evaluated the performance of the proposed method with RetinaNet [51], CenterNet [19], YOLOv5, YOLOv8, and GCC-YOLO [4]. Table 1 shows the quantitative experimental results for the various methods, and Figure 6 shows the visualization results. Furthermore, since lightweight and lower-complexity models are crucial for manufacturing lines, we also compare the stability and complexity of the proposed method with other state-of-the-art methods. The results are shown in Table 2.

The quantitative results show that although RetinaNet can obtain relatively good performance, it requires a large number of computational resources, so it cannot be directly deployed to the manufacturing line for PCB defect detection. YOLO still needs to be further improved for the detection of small targets, especially when the target has a low contrast or is surrounded by noise, such as mouse bite. CenterNet can effectively detect targets of different sizes as it predicts the center point of the target by using a heatmap of key points. However, its high computational complexity is still a challenge for deployment in production lines. The proposed algorithm achieves comparable or even better performance than the other algorithms using a fewer number of parameters and with lower FLOPs.

Furthermore, the manually labeled bounding boxes and the prediction bounding boxes of several approaches are displayed in Figure 6. For the same series of models, e.g., Retinanet-ResNet18, Retinanet-ResNet34, and Retinanet-ResNet50, only the model with the smallest parameter is illustrated. So, only the detection results of Retinanet-ResNet18 are illustrated in Figure 6. The results show that the proposed algorithm is able to accurately detect all types of defects despite the small size of the defect targets.

### 4.4. Ablation Study

In this section, to confirm that each component of the proposed approach is beneficial, we decompose the proposed algorithm into modules and conduct a detailed ablation study.

**Darknet53-PANet**: In this paper, we design the LGCL-CenterNet based on the original CenterNet using ResNet18 as the backbone (Baseline in Table 3) to effectively and accurately detect PCB defects. It can be seen that the existing CenterNet model requires large parameters and high computational effort which do not meet the practical manufacturing line requirements. The YOLO series of algorithms has become one of the important algorithms in the field of target detection with its real-time, efficient network structure, multi-scale feature fusion, and end-to-end training, and has achieved good results in many practical applications. However, these algorithms cannot efficiently detect smaller targets such as PCB defects. To balance efficiency and performance, the backbone network of YOLOv8 and the neck network are used to replace the backbone network of CenterNet to reduce the number of parameters as well as FLOPs. After the backbone network was replaced, Model 1 slightly enhanced detection performance and drastically decreased the number of parameters and FLOPs.

**DA**: Data augmentation (DA) is a technique that transforms and expands the original data during the training process, aiming to increase the diversity of the training data and improve the generalization ability and robustness of the model. The number of PCB datasets used in this paper is small, and the use of data enhancement techniques can effectively increase the amount of data to reduce overfitting. It can be seen from Model2 that the performance improves significantly after using data enhancement.

**Remove P5:** The high-dimensional features (P5) will increase the computational complexity of the model, and continuous downsampling for small target detection is not friendly. Thus, this section conducts an ablation study by removing the P5 dimensional features to verify its effectiveness. We find that P5 dimensional features do not significantly help PCB defect detection or even cause model performance degradation due to too many parameters and insufficient training data (Model2), and greater performance can be achieved by not using P5 (Model3) dimensional features.

**BRA** [47]: Improved bi-level routing attention (BRA) achieves more flexible computational allocation and content awareness by using dynamic sparse attention, which retains fine-grained detail information while reducing computational complexity. By introducing it into the backbone network to replace the spatial pyramid pooling-fast (SPPF) module to integrate the global features, as shown in Table 3, it can be demonstrated that the global sparse attention module (BRA) is able to further improve the detection of PCB defects and reduce the computation complexity by comparing Model3 and Model4. Since PCB defects are not only small but sometimes dense, some defect targets may be filtered out during the continuous pooling process of the SPPF.

**Clo** [48]: The Clo module uses a two-branch partition structure to extract high-frequency local features and low-frequency global features for downstream tasks, respectively. Model5 explores the use of the Clo module instead of the SPPF module to extract local as well as global features. The module demonstrates that local high-frequency features can also improve the performance of PCB defect detection.

**LGT**: In order to efficiently aggregate the local and global information extracted from the backbone network, based on the BRA and Clo modules, this paper designs a two-branch lightweight vision transformer module with local and global attention, named LGT block. Comparing Model3 with Model6, it can be seen that this module improves performance without increasing the number of parameters too much. Furthermore, by comparing it with Model5, it can be concluded that the possible reason for the better performance of LGT is that queries in different semantic areas actually focus on different key-value pairs. Hence, it might not be the best idea to make all queries focus on the whole feature map. In addition, the local high-frequency feature extraction branch is not only capable of weighted fusion of the feature maps to selectively enhance the local feature representations but also enhances the feature extraction capability of the model for PCB defects.

**LWH**: In order to further reduce the FLOPs and parameters, this paper simplifies the head network of CenterNet by using depthwise separable convolution. Model7 maintains model performance while drastically reducing the number of parameters and FLOPs.

**1/2. Size**: In addition, this paper uses the original image as input for training and testing. In practical manufacturing lines, downsampling can also be used to further speed up the inference.

## 5. Conclusions

Printed circuit board (PCB) defect detection and accurate positioning are crucial components of quality control in PCB manufacturing. Currently, the PCB industry is still using traditional inspection tools for quality inspection, which requires considerable manual operation and cannot ensure efficient and stable quality inspection. In this paper, an efficient real-time PCB defect detection algorithm is proposed based on CenterNet. Specifically, a lightweight backbone network is designed to replace the original residual network of CenterNet, and to aggregate the global and local features extracted from the backbone network, a two-branch lightweight real-time visual transformer block combining the local and global context information is designed, LGT block, for further fusion and processing of the extracted features to improve the expressive capability of the model. Then, PANet is introduced to aggregate multi-scale features extracted from the backbone network to reduce shallow feature loss. Finally, to further reduce the computational complexity, this paper designs a lightweight prediction head based on depthwise separable convolution to further integrate the extracted features to output the final PCB defect location and category.

Due to the small number of manually labeled PCB defects, this paper first uses coco data to pre-train the proposed model, and then fine-tune the model. However, there are differences in texture between natural images and PCB images, so the pre-trained model using coco is not necessarily suitable for PCB defect detection. In practice, PCB images are relatively easy to obtain, but image annotation requires substantial manual resources, so we explore the use of self-supervised algorithms [19] to obtain a pre-trained model, i.e., training based on PCB data, which can efficiently extract PCB texture features, and then use a small amount of labeled data for fine-tuning to obtain the final PCB defect detection model.

## Figures and Tables

**Table 1:** Average Precision (AP) Achieved with IoU = 0.50 for PCB Defect Classification Across Defect Types.

Method			AP_50_(%)				mAP@50 (%)	mAP@75 (%)	mAP@50:95 (%)	mAP^S^@50:95 (%)
	Sh	Mh	Sp	Mb	Sc	Oc				
YOLOv5s	0.988	0.9931	0.9688	0.9722	0.9814	0.9828	0.9810	0.442	0.514	0.457
YOLOv8n	0.9672	0.9815	0.9898	0.9444	1.0	0.969	0.9735	0.488	0.516	0.454
Retinanet-ResNet18	0.9424	0.9815	0.9936	0.9729	0.9994	0.9886	0.9797	0.387	0.493	0.443
CenterNet-ResNet18	0.9452	0.9726	0.943	0.9848	1.0	0.9634	0.9682	0.4	0.482	0.5
YOLOv5m	0.9893	1.0	0.9739	0.967	1.0	0.9706	0.9835	0.477	0.53	0.455
YOLOv8s	0.9774	0.968	0.9795	0.9715	1.0	0.9883	0.9808	0.421	0.501	0.544
Retinanet-ResNet34	0.9804	0.9918	0.9951	0.9735	0.981	0.966	0.9813	0.42	0.494	0.482
CenterNet-ResNet34	0.9756	1	0.9198	0.9687	0.9752	0.972	0.9686	0.398	0.478	0.459
YOLOv5l	0.9907	0.9964	0.9858	0.9693	1.0	0.9809	0.9872	0.499	0.532	0.461
YOLOv8m	0.9663	0.9861	0.9708	0.9672	1.0	**0.9915**	0.9802	0.423	0.499	0.596
Retinanet-ResNet50	0.9736	0.9948	0.9937	0.9507	0.994	0.9786	0.9809	0.42	0.5	0.526
CenterNet-ResNet50	0.9658	1.0	0.9729	0.9476	1	0.9734	0.9766	0.424	0.494	0.469
GCC-YOLO	0.9491	0.9834	0.9814	**0.9842**	0.9995	0.9845	0.9804	0.478	0.506	0.483
Ours	**0.9930**	**1.0**	**0.9972**	0.9675	0.9997	0.9796	**0.9895**	**0.516**	**0.529**	**0.602**

**Table 2:** Comparison of Model Complexity and Stability of Advanced Object Detection Algorithms.

Method	FLOPs	Paras (M)	FPS
YOLOv5s	190.223G	7.077	49.76
YOLOv8n	94.418G	3.012	65.28
Retinanet-ResNet18	1.436T	19.875	14.89
CenterNet-ResNet18	648.185G	14.128	49.35
YOLOv5m	583.382G	21.077	26.26
YOLOv8s	329.994G	11.138	44.79
Retinanet-ResNet34	1.785T	29.983	13.51
CenterNet-ResNet34	996.870G	24.236	34.74
YOLO V5l	1.320T	46.658	17.31
YOLO V8m	910.626G	25.860	25.8
Retinanet-ResNet50	1.904T	36.434	12.7
CenterNet-ResNet50	1.336T	33.009	20.26
GCC-YOLO	202.972G	6.812	35.29
Ours	**45.316G**	**0.542**	51.18

**Table 3:** Ablation Experiment Results.

Model	Darknet53-PANet	DA	Remove P5	BRA	CLo	LGT	LWH	1/2 Size	FLOPs (G)	Paras (M)	mAP [IoU = 0.5] (%)
Baseline									648.185	14.128	0.9682
Model1	√								134.148	2.425	0.9711
Model2	√	√							134.148	2.425	0.9811
Model3	√	√	√						110.273	0.643	0.9832
Model4	√	√	√	√					109.381	0.620	0.9839
Model5	√	√	√		√				111.177	0.680	0.9843
Model6	√	√	√			√			109.952	0.649	0.9863
Model7	√	√	√			√	√		45.316	0.542	0.9895
Model8	√	√	√			√	√	√	11.329	0.542	0.9832

## Data Availability

This study used open-access online datasets.

## Supplementary Materials

The following supporting information can be downloaded at: https://www.mdpi.com/article/10.3390/s24144729/s1, Figure S1: Detection results of different object detection algorithms.
