# Supplementary material for: Local and Global Context-Enhanced Lightweight CenterNet for PCB Surface Defect Detection
Source: Sensors (Basel). 2024 Jul 21;24(14):4729. doi: 10.3390/s24144729 (PMC11281245; doi:10.3390/s24144729)

# Supplementary Material
# Detection results of different object detection algorithms

1. Mouse bite

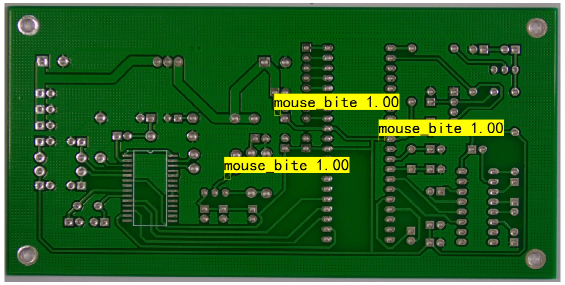


Ground truth

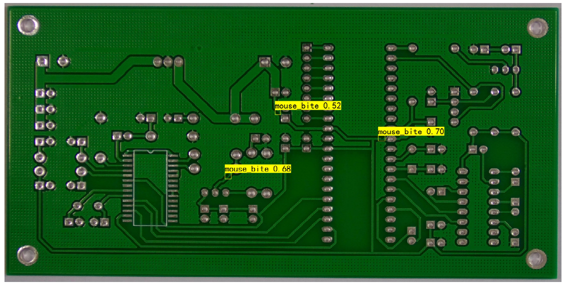

GCC-YOLO

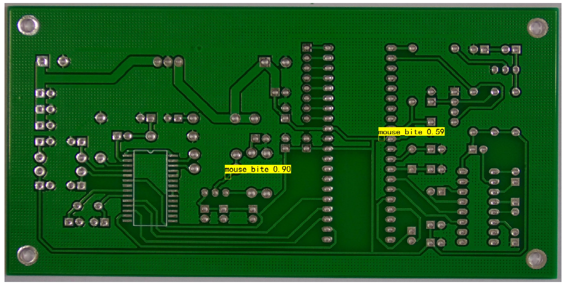

RetinaNet-ResNet18

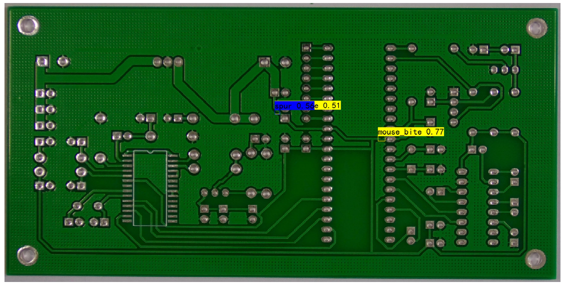

YOLOv8n

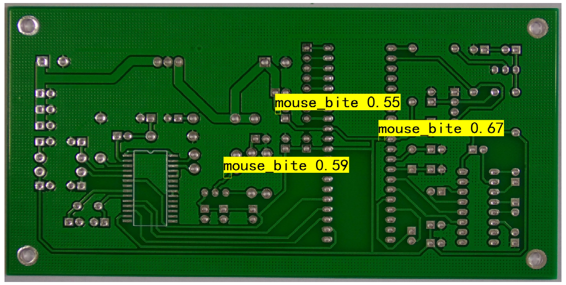


CenterNet-ResNet18

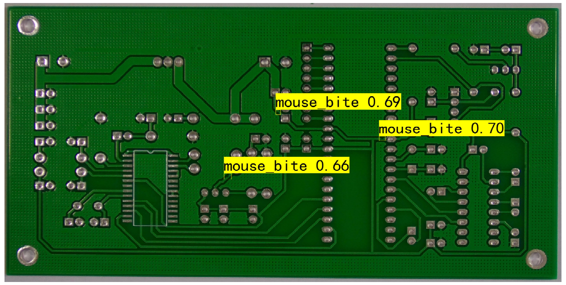


Ours

2. Open circut

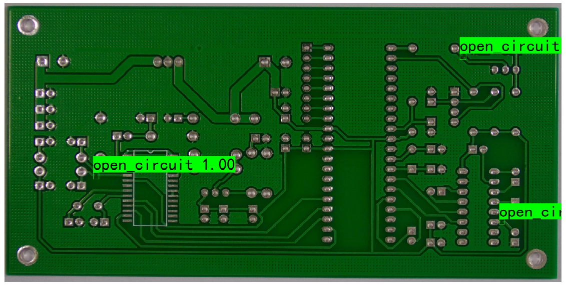


Ground truth

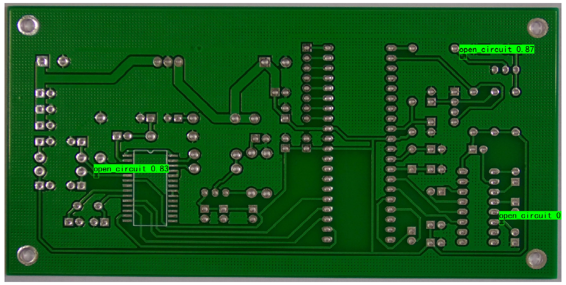

GCC-YOLO

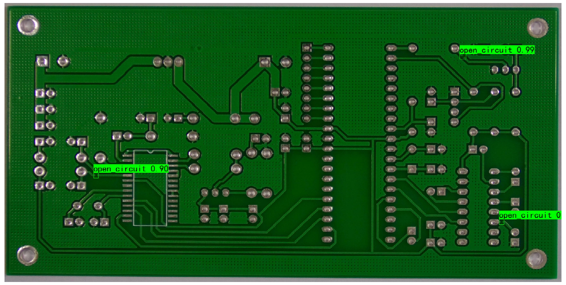

RetinaNet-ResNet18

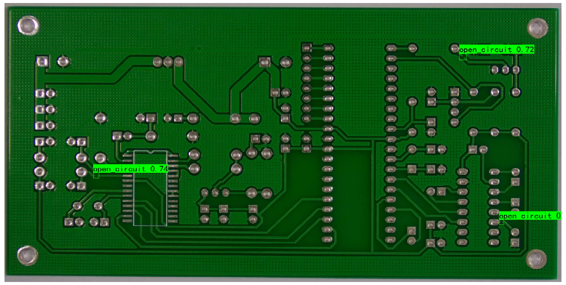

YOLOv8n

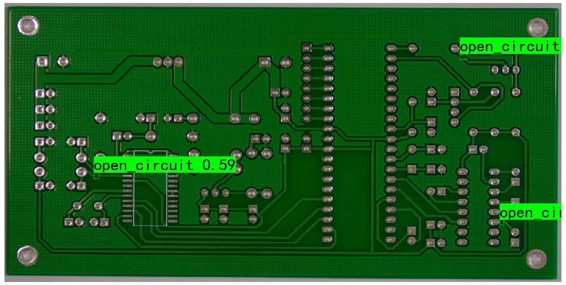


CenterNet-ResNet18

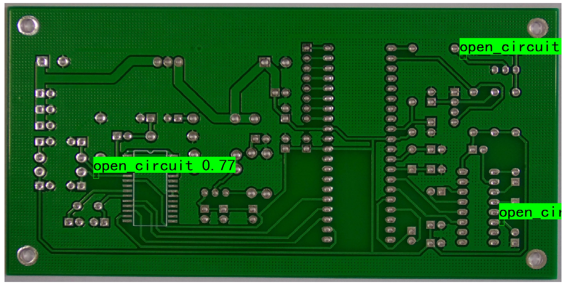


Ours

3. Spurious copper

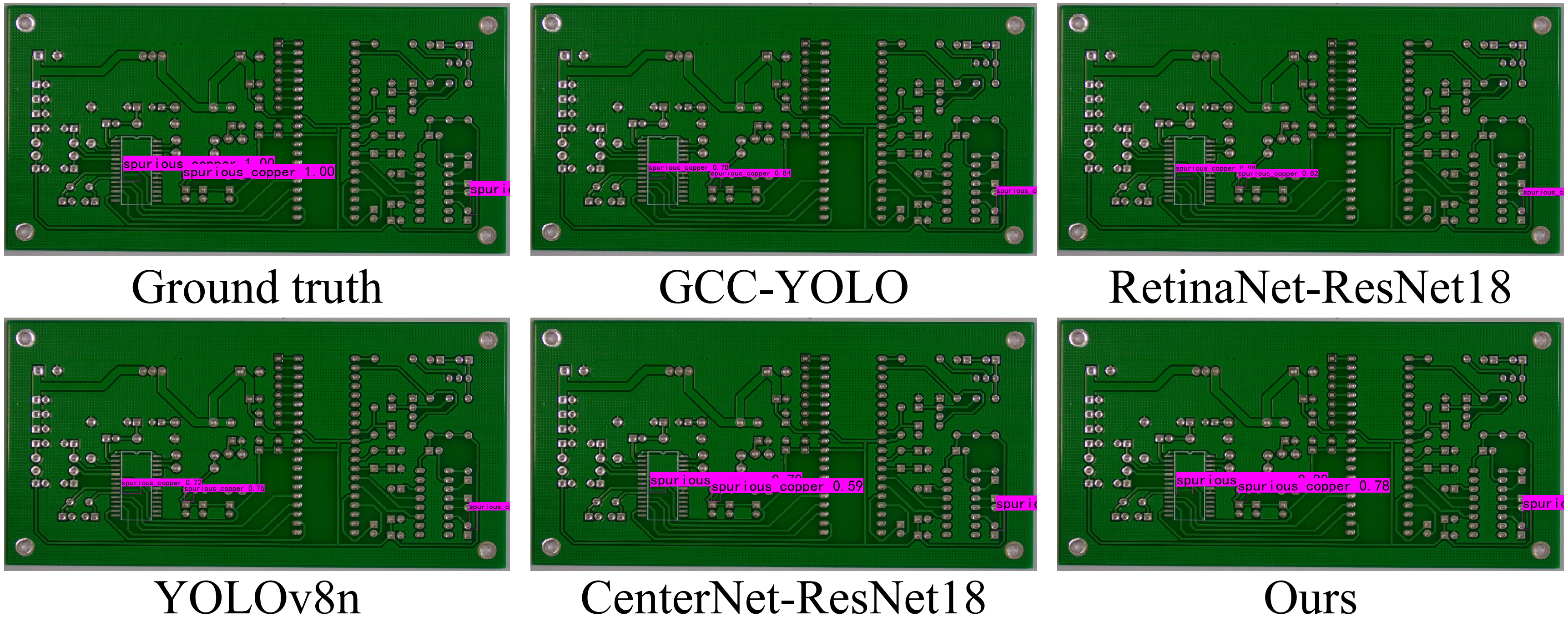

4. Missing hole

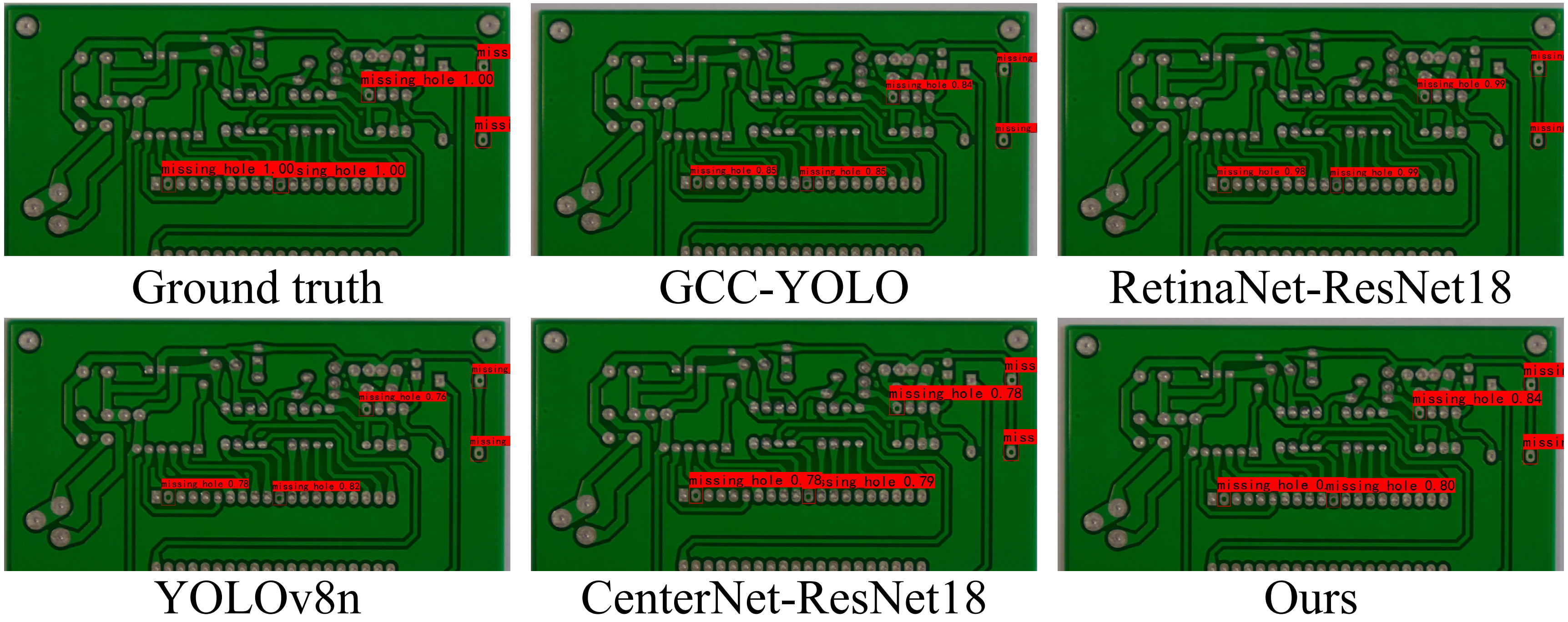

Supplement: Supplementary file 1 [file sensors-24-04729-s001.zip › sensors-3061216-supplementary.pdf]
